# Crystal structure and Hirshfeld surface analysis of 3-acetyl-11-keto-β-boswellic acid

**DOI:** 10.1107/S2056989026005360

**Published:** 2026-05-29

**Authors:** B. Dinesh, H. N. Shafiulla, J. C. Shwetha, A. H. Udaya Kumar, N. K. Lokanath, K. Anand Solomon

**Affiliations:** a Department of Phytochemistry, Greenspace Herbs, Brigade Twin Towers, Yeshwanthpur, Bengaluru-560022. Karnataka, India; b Department of Chemistry Sri Sathya Sai University for Human Excellence Nallakadirenahalli 561211 India; c Department of Physics, Seshadripuram Institute of Technology, Kadakola industrial area, Mysore 571311, Karnataka, India; d Department of Studies in Physics University of Mysore, Mysuru 570006 Karnataka India; Universidade Federal do ABC, Brazil

**Keywords:** 3-acetyl-11-keto-β-boswellic acid, hydrogen bonds, Hirshfeld surface analysis, crystal structure

## Abstract

Acetyl-11-keto-β-boswellic acid, C_32_H_48_O_5_, a penta­cyclic triterpenoid from *Boswellia serrata*, exhibits notable anti-inflammatory and pharmacological activities. The compound crystallizes in the ortho­rhom­bic space group *P*2_1_2_1_2 and exhibits a rigid penta­cyclic framework with eleven stereogenic centers. The cyclo­hexane rings adopt near-ideal chair conformations with minimal steric strain. The crystal packing is governed by O—H⋯O hydrogen bonds, forming zigzag chains along the [100] direction and extending into a three-dimensional network and is further consolidated by van der Waals inter­actions. Hirshfeld surface analysis shows dominant H⋯H contacts (91.5%), highlighting the importance of van der Waals forces, while H⋯O/O⋯H contacts provide localized stabilization.

## Abstract 2

The title compound crystallizes in the ortho­rhom­bic space group *P*2_1_2_1_2 and exhibits a rigid penta­cyclic framework with eleven stereogenic centres. The cyclo­hexane rings adopt near-ideal chair conformations with minimal steric strain. The crystal packing is governed by O—H⋯O hydrogen bonds, forming zigzag chains along the [100] direction and extending into a three-dimensional network and is further consolidated by van der Waals inter­actions.

## 1. Chemical context

Acetyl-11-keto-β-boswellic acid (AKBA) is a natural compound isolated from the dried gum resin of *Boswellia Serrata*. It belongs to ursane-type penta­cyclic triterpene class, containing fused cyclo­hexane rings. The mol­ecule bears several oxygen-containing functional groups, including carb­oxy­lic acid, ketone, and acetyl substituents (Park *et al.*, 2002 ▸), which contribute to inter­molecular inter­actions within the crystal. The cardioprotective activity of these compounds has been recorded (Teng *et al.*, 2024 ▸). AKBA exhibits inhibitory effects on cultured human umbilical vascular endothelial cells (Shen *et al.*, 2015 ▸), and also exhibits anti-proliferative (Li *et al.*, 2022 ▸), and anti-dermatitis (Tsai *et al.*, 2022 ▸) activity. It functions as a selective inhibitor of 5-lipoxygenase, a key enzyme in leukotriene biosynthesis, with demonstrated anti-inflammatory and anti-arthritic activity (Sailer *et al.*, 1996 ▸). The mol­ecule’s lipophilic nature, inherent to its steroid-like scaffold, presents formulation challenges but also enables membrane permeability and inter­action with hydro­phobic enzyme active sites (Lindner *et al.*, 2026 ▸).
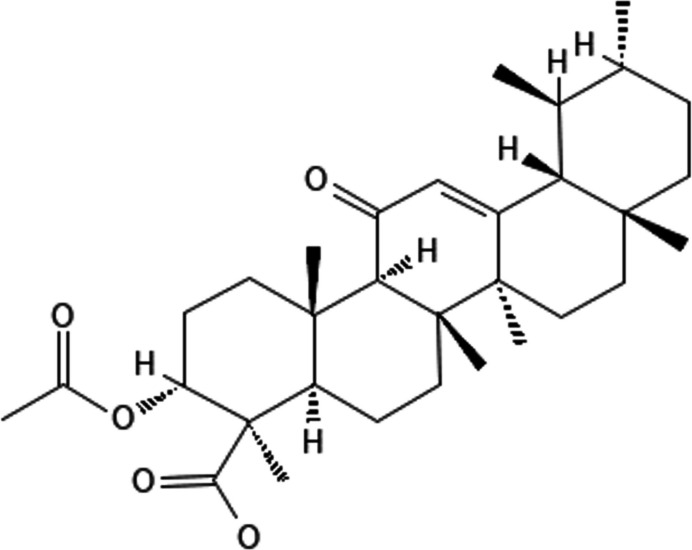


## 2. Structural commentary

**Figure 1:**
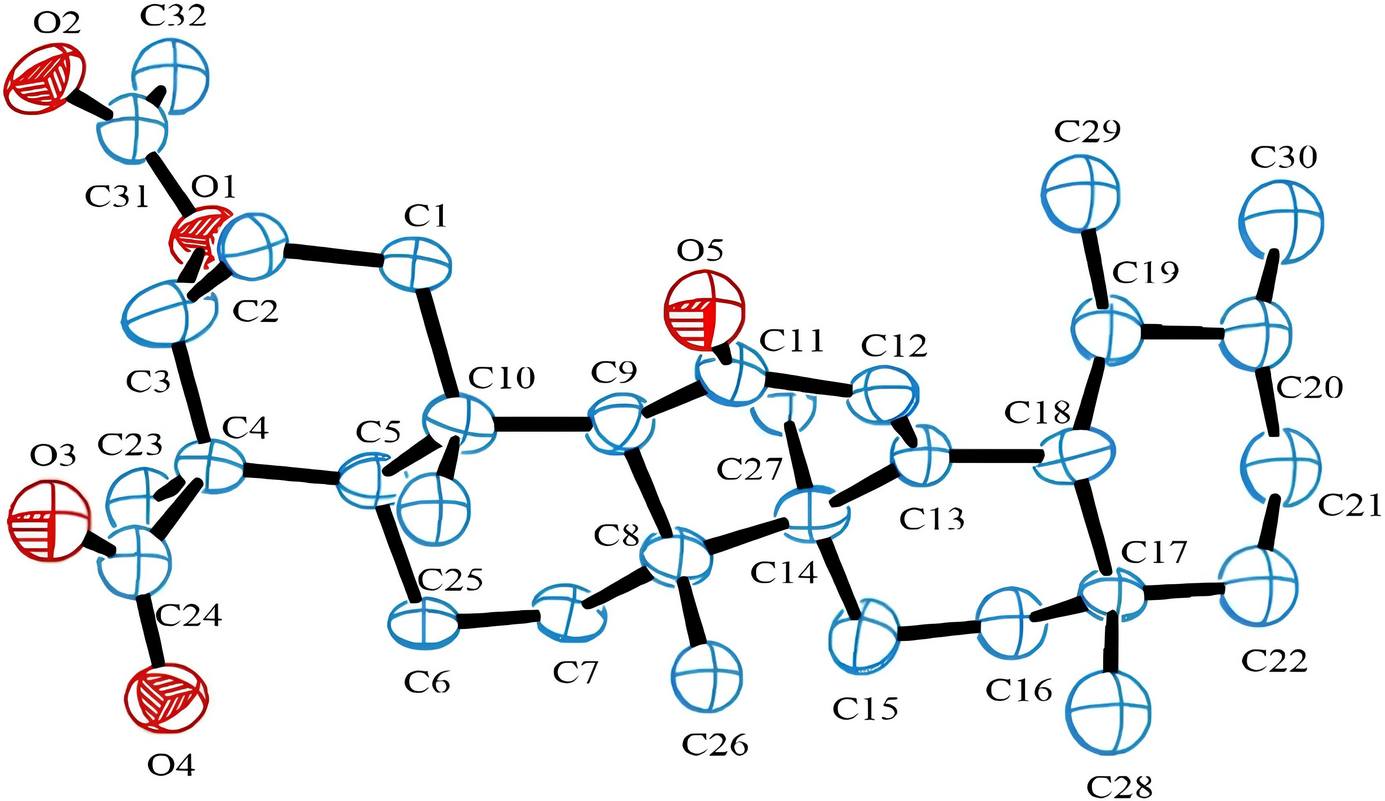
Acetyl-11-keto-β-boswellic acid (AKBA) showing the atomic numbering scheme. Displacement ellipsoids are drawn at the 30% probability level, and hydrogen atoms are omitted for clarity.

The title mol­ecule (Fig. 1 ▸) crystallizes in ortho­rhom­bic system, space group *P*2_1_2_1_2, with four mol­ecules in the unit cell (*Z* = 4). AKBA possesses eleven stereogenic centres – C3, C4, C5, C8, C9, C17, C18, C20 in the *R* configuration and C10, C14, C19 in the *S* configuration. The acet­oxy group is at the α position (Ito *et al.*, 2025*a* ▸). This forces a planar arrangement of the six atoms O3/C9/C11–C14, with deviations from the least-square plane being less than 0.036 Å. The five six-membered rings are fused in such a manner that the C—C bonds occupy equatorial positions, except for the C18—C19 bond, which is in an axial position with respect to the C13–C18 ring. As a consequence, all cyclo­hexane rings form a sheet-like structure, apart from the C17–C22 ring, which is oriented roughly orthogonal to this plane [C13—C18—C17—C22 = 174.78 (4)°]. A puckering analysis of conformational characteristics of the fused six-membered rings according to the Cremer-Pople approach was qu­anti­tatively evaluated and revealed that the crystal structure exhibits mainly distorted chair-like conformational forms.

For Ring (1) (C1–C5/C10), the puckering parameters are *Q* = 0.541 (4) Å, θ = 5.9 (4)° and φ = 320 (4)° indicating an almost ideal chair conformation. The very small θ value together with the dominant *Q*(3) contribution [0.538 (4) Å] confirms that the ring closely resembles a classical cyclo­hexane chair geometry. This assignment is further supported by the alternating torsion angles ranging from −55.2 (4) to 55.1 (4)° and by the Evans–Boeyens conformational analysis, which describes the ring as very similar to a C-form.

Ring (2) (C5–C10) also adopts a chair conformation with puckering parameters *Q* = 0.547 (4) Å, θ = 13.4 (4)° and φ = 25.3 (16)°. Although the θ value is slightly larger than that observed for Ring (1), the dominant *Q*(3) term [0.532 (4) Å] clearly establishes a chair-type geometry with minor distortion. The observed torsion angles, varying between −63.3 (4) and 57.9 (4)°, are consistent with a puckered cyclo­hexane framework.

In contrast, Ring (3) (C8/C9/C11–C14) exhibits a significantly distorted conformation arising from the presence of *sp*^2^-hybridized atoms within the ring skeleton. The Cremer–Pople parameters [*Q* = 0.563 (4) Å, θ = 54.4 (4)°, φ = 6.5 (5)°] indicate a conformation inter­mediate between a half-chair and twist-boat geometry. The comparatively large *Q*(2) value [0.458 (4) Å] demonstrates a substantial deviation from an ideal chair form, while the reduced average torsion angle of approximately 40.7° further supports the presence of conformational distortion induced by partial unsaturation.

Ring (4) (C13–C18) adopts an inverted chair conformation, as evidenced by the puckering parameters *Q* = 0.517 (4) Å, θ = 160.4 (4)°, and φ = 41.8 (14)°. The θ value approaching 180° is characteristic of an inverted-chair geometry, while the dominant negative *Q*(3) component [−0.487 (4) Å] further substanti­ates this assignment. The alternating torsion angles observed within the ring are typical of a puckered six-membered ring adopting a chair-like arrangement with slight distortion due to substitution effects.

Similarly, Ring (5) (C17–C22) displays a near-ideal inverted-chair conformation with puckering parameters *Q* = 0.528 (5) Å, θ = 174.0 (5)° and φ = 27 (5)°. The negligible *Q*(2) contribution together with the dominant negative *Q*(3) value [−0.525 (5) Å] confirms the highly stable chair geometry. The Evans–Boeyens analysis also classifies this ring as being very close to a C-form conformation.

The six-membered ring is composed of *sp*^3^-hybridized atoms, with normal bond lengths between carbon atoms (1.5416 Å) and bond angles close to tetra­hedral, suggesting a strain-free saturated ring system. The presence of alternating torsion angles, approximately ±50°, together with small deviations from planarity, is consistent with a puckered ring conformation

## 3. Supra­molecular features and Hirshfeld surface analysis

**Figure 2:**
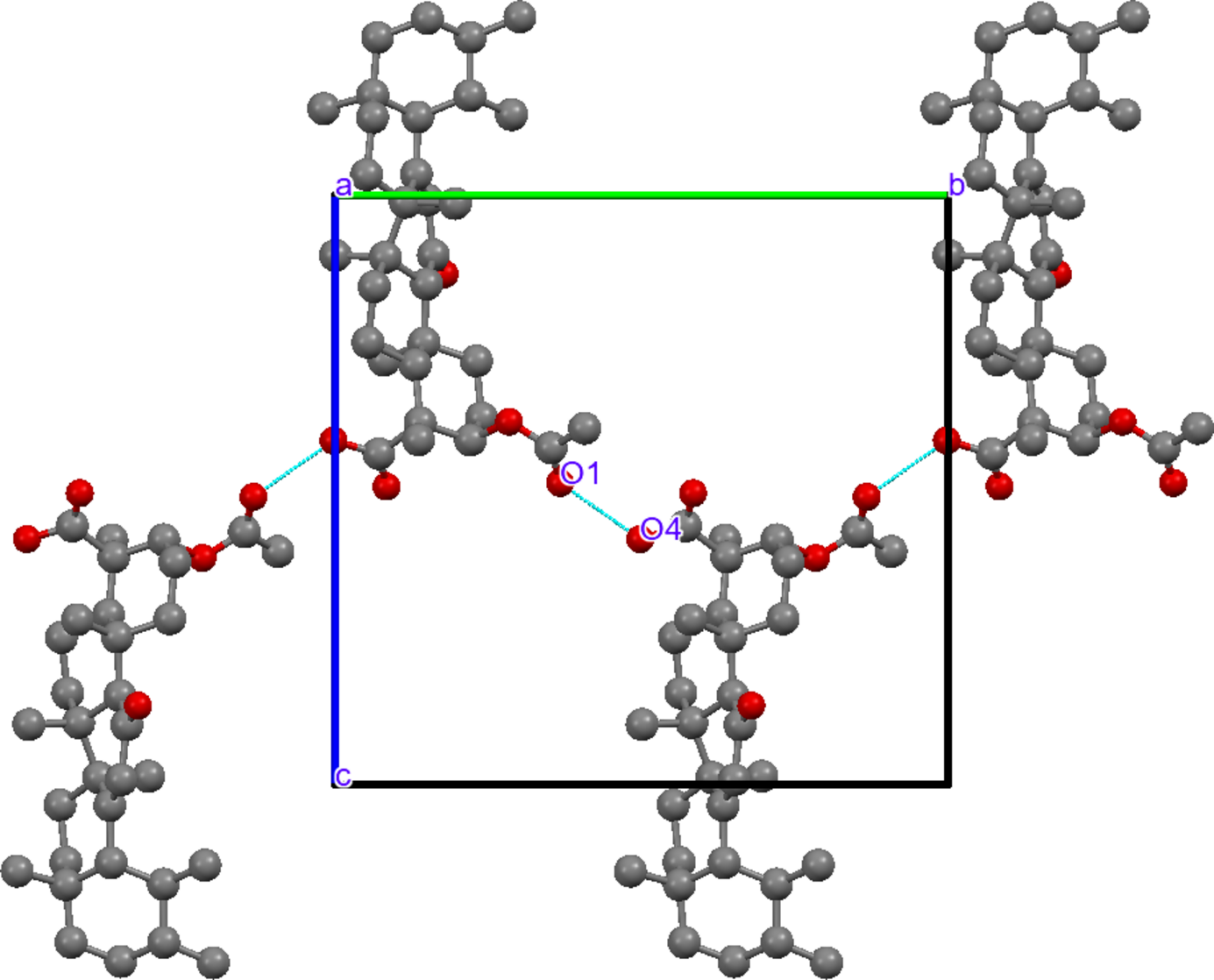
Crystal packing of acetyl-11-keto-β-boswellic acid (AKBA) viewed along the *c* axis, illustrating the mol­ecular arrangement within the unit cell. Inter­molecular inter­actions and packing orientation are highlighted, and the unit-cell boundaries are shown.

In the extended structure of AKBA, the mol­ecules are linked by O—H⋯O hydrogen bonds (Table 1 ▸) from the carb­oxy­lic acid OH group to the carbonyl oxygen atom, forming a *C*4 zigzag chain propagating along the [100] direction (Fig. 2 ▸). The hydro­carbon framework of the mol­ecule forms hydro­phobic regions, while the oxygenated functional groups participate in hydrogen bonding, leading to an organized packing arrangement within the ortho­rhom­bic structure.

The Hirshfeld surface mapped over *d*_norm_ for AKBA reveals localized red regions corresponding to short inter­molecular contacts involving oxygen-containing functional groups. These red spots are associated mainly with close H⋯O/O⋯H inter­actions involving the acetyl, keto and carb­oxy­lic oxygen atoms, indicating contacts shorter than the sum of the corresponding van der Waals radii. White areas correspond to contacts close to van der Waals separations, whereas blue regions represent distances longer than the van der Waals radii and therefore weaker inter­molecular contacts.

**Figure 3:**
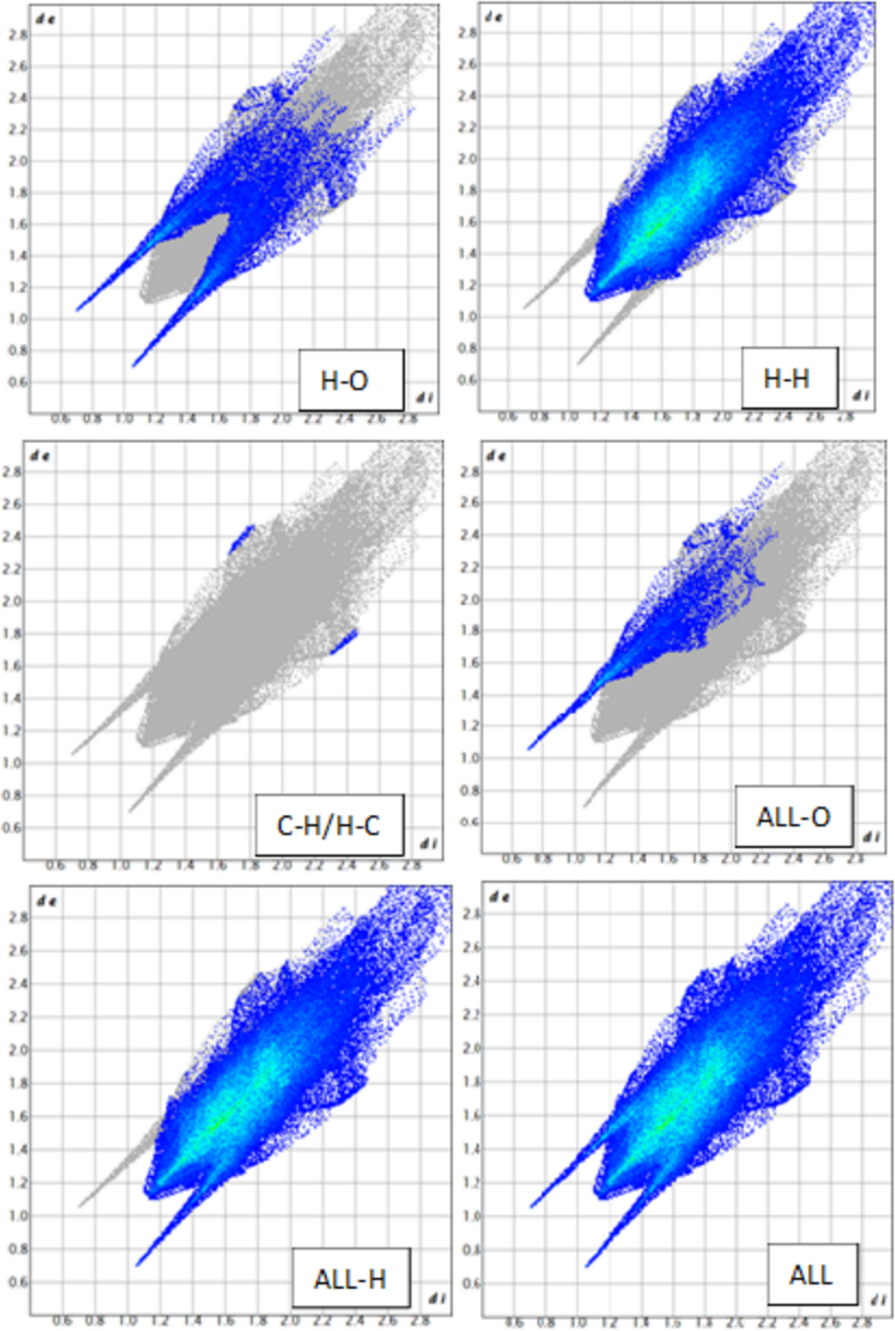
Hirshfeld surface fingerprint plots for acetyl-11-keto-β-boswellic acid (AKBA) showing the contributions of different inter­molecular contacts: H⋯O/O⋯H, H⋯H, C⋯H/H⋯C, and all contacts (ALL and ALL–H). The blue regions represent the specific inter­actions within the mol­ecule, while the grey areas correspond to the overall fingerprint plots. These plots highlight the dominant role of H⋯H and H⋯O inter­actions in consolidating the crystal packing.

**Figure 4:**
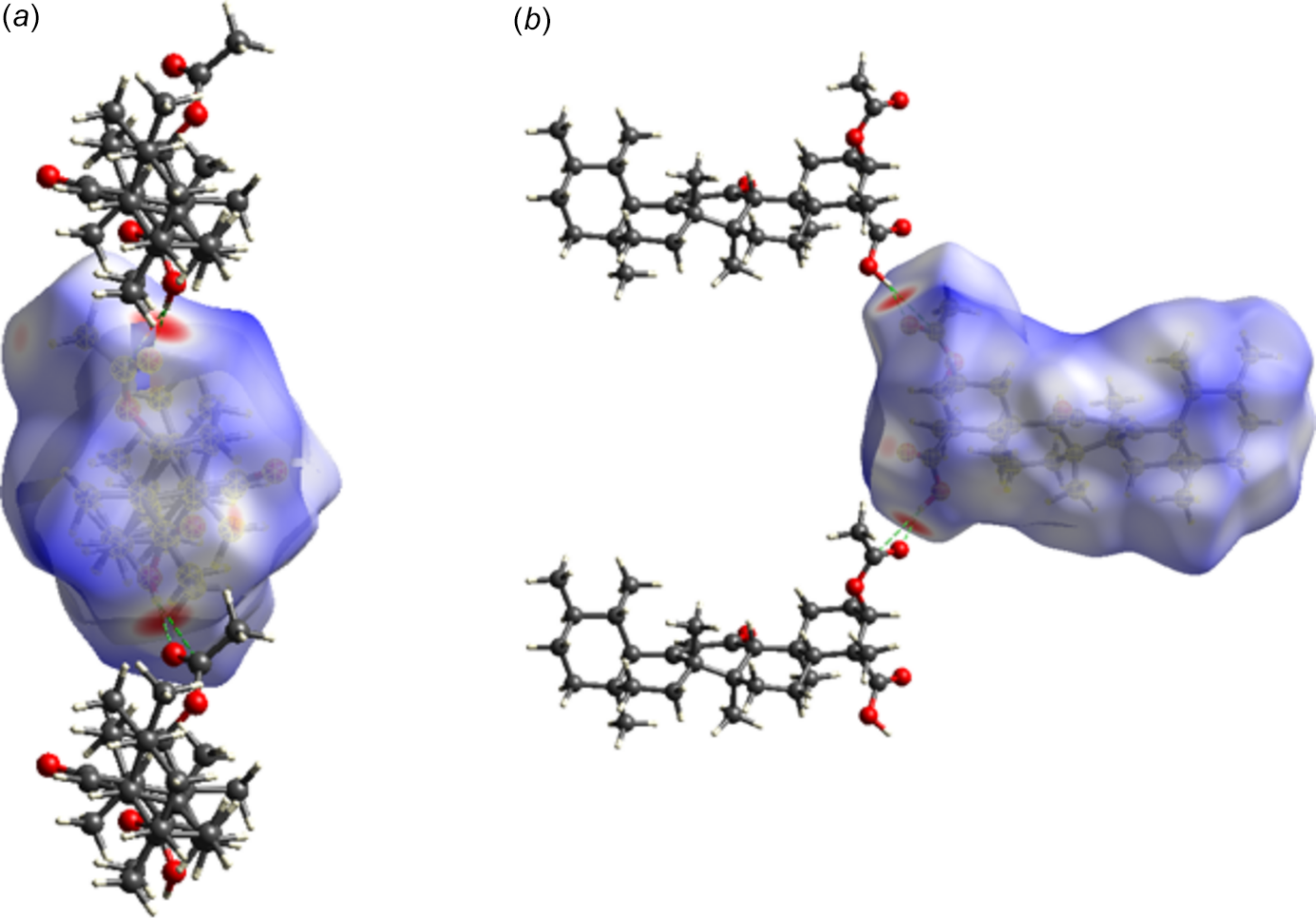
Hirshfeld surfaces mapped over *d*_norm_: (*a*) front view and (*b*) side view showing short inter­molecular contacts as red regions.

**Figure 5:**
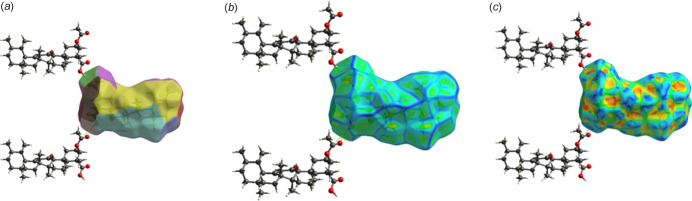
Hirshfeld surfaces mapped over (*a*) fragment patch, (*b*) curvedness and (*c*) shape-index, showing neighbouring mol­ecular fragments and local surface features associated with the inter­molecular packing.

The Hirshfeld surface area was calculated as 509.74 Å, with a surface volume of 755.63 Å. The globularity value of 0.787 indicates a compact but slightly elongated mol­ecular envelope consistent with the rigid penta­cyclic triterpenoid framework, whereas the asphericity value of 0.187 reflects moderate anisotropy arising from the extended substituent groups. Two-dimensional fingerprint plots show that H⋯H contacts dominate the crystal packing, contributing 91.5% of the total Hirshfeld surface. The broad central distribution in the H⋯H fingerprint plot reflects extensive hydro­carbon–hydro­carbon inter­actions arising from the large penta­cyclic triterpenoid framework, confirming that van der Waals inter­actions are the principal consolidating force in the crystal. H⋯O/O⋯H contacts contribute 8.3% of the Hirshfeld surface and appear as distinct sharp spikes in the fingerprint plot. These spikes correspond to short inter­molecular contacts involving oxygen acceptor atoms and indicate weak C—H⋯O inter­actions that provide localized consolidation around the polar functional groups. The reciprocal O⋯H/H⋯O contribution amounts to 17.6% when reciprocal contacts are considered, reflecting the combined donor–acceptor inter­action environment surrounding the oxygen atoms. C⋯H/H⋯C contacts contribute only 0.2% of the surface and are represented by small isolated wing-like regions, indicating that weak hydro­phobic carbon–hydrogen contacts make only a minor contribution to crystal packing. C⋯O/O⋯C inter­actions are negligible (0.1–0.2%), showing that direct carbon­yl–carbon contacts are not significant in the present crystal structure (Fig. 3 ▸). The Hirshfeld surface mapped over *d*_norm_ displays several small bright-red spots, corresponding to weak and longer range inter­actions that contribute to the consolidation of the packing (Fig. 4 ▸). The fragment patch highlights key neighbouring mol­ecular inter­actions contributing to the crystal packing. The curvedness map indicates predominantly flat regions, suggesting the absence of significant π–π stacking inter­actions. The shape-index surface shows complementary patterns, confirming localized inter­molecular contacts such as hydrogen bonding (Fig. 5 ▸).

## 4. Database survey

**Figure 6:**
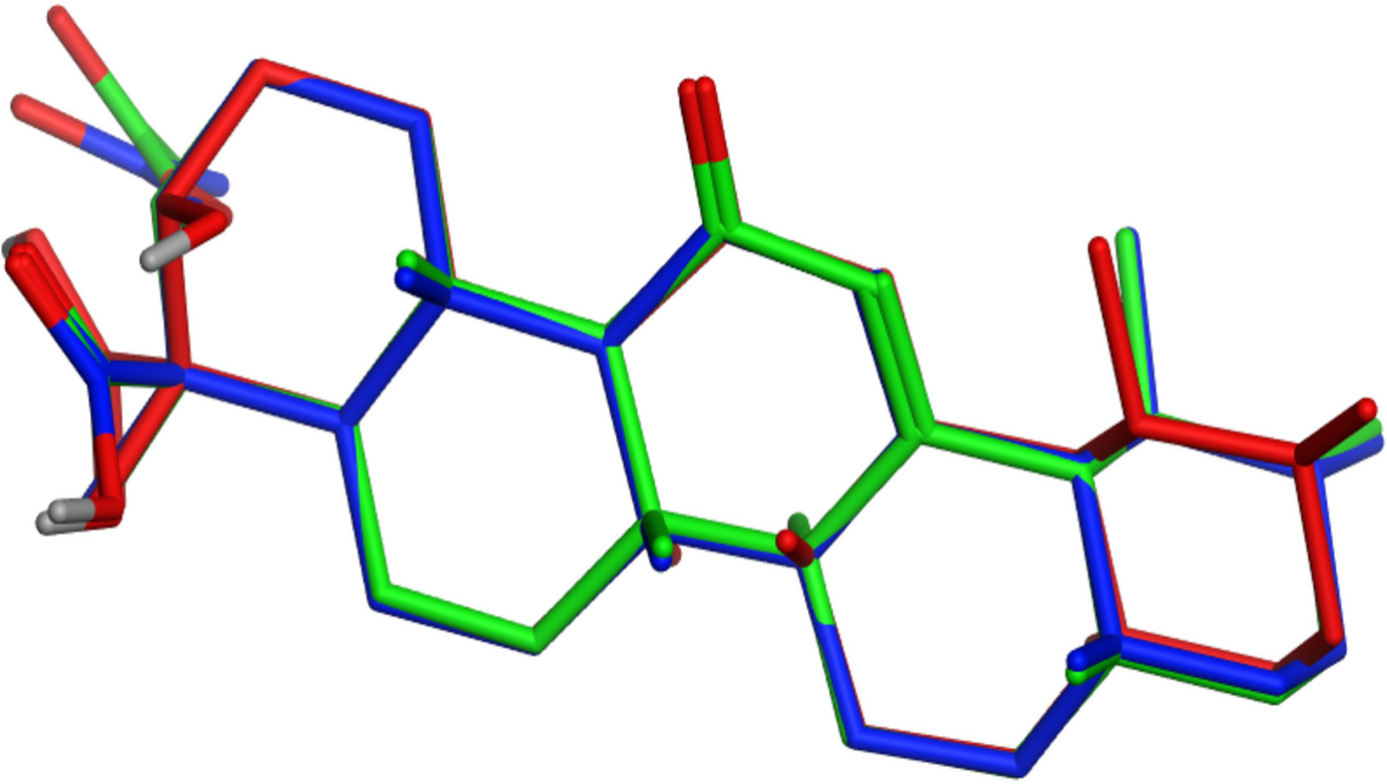
Superimposed mol­ecular structures of boswellic acid derivatives showing conformational differences: acetyl β-boswellic acid (blue), 11-keto-β-boswellic acid (red) and acetyl-11-keto-β-boswellic acid (green).

A search of the Cambridge Structural Database (CSD, version 6.00 update of May 2025; Groom *et al.*, 2016 ▸) for compounds containing the boswellic acid skeleton shows that only a limited number of crystal structures of boswellic acid derivatives have been reported. These include β-boswellic acid, acetyl-β-boswellic acid, and 11-keto-β-boswellic acid derivatives (Majeed *et al.*, 2024 ▸; Ito *et al.*, 2025*b* ▸). These compounds share the same penta­cyclic triterpenoid framework composed of fused cyclo­hexane rings, adopting stable chair or slightly distorted chair conformations. In all the structures, the stereochemistry at the ring junctions remains conserved, confirming that chemical modifications at peripheral positions do not significantly alter the rigid triterpenoid backbone. Comparison with previously reported boswellic acid structures indicates that the overall structure conforms with those with other derivatives of the boswellic acid family (Fig. 6 ▸). However, substitution at the C3 and C11 positions significantly influences the inter­molecular inter­actions and crystal packing (Al-Harrasi *et al.*, 2018 ▸). In β-boswellic acid, the hydroxyl group at C3 can participate as a hydrogen-bond donor, frequently forming inter­molecular O—H⋯O hydrogen bonds that contribute to crystal cohesion. In addition, the compound contains an acetyl group at C3, which replaces the hydroxyl donor with an ester carbonyl acceptor, thereby reducing classical hydrogen-bonding capability (Khaafi & Javadi, 2023 ▸)

## 5. Synthesis and crystallization

Fresh frankincense resin lumps of 6 g were ground to a fine, uniform powder with a mortar and pestle. Primarily, 100 mg of powdered resin was transferred into a 2 mL reaction tube and 1 mL of extraction solvent (methanol: 1% aqueous formic acid, 65:35 *v*/*v*) was added. To promote efficient release of triterpenes, the suspension was sonicated at 298 K for 10 minutes and then centrifuged at 14,000 r.p.m. for 5 minutes; the clear supernatant was deca­nted and reserved. The pooled AKBA-rich supernatent was subjected to flash chromatography on silica (230–400 mesh) using *n*-hexane (solvent A) and ethyl acetate (solvent B) with a gentle gradient (flow ≃ 1 mL min^−1^), guided by TLC. Fractions eluting at ∼40–50% EtOAc were concentrated under reduced pressure at low bath temperature. Residual non-polar impurities were removed by washing the concentrate with cold *n*-hexane; the remaining polar residue was dissolved in minimum hot aceto­nitrile and allowed to cool slowly to room temperature (Gupta *et al.*, 2021 ▸; Lauss *et al.*, 2024 ▸). The resulting pale-white crystals were collected by filtration and dried under vacuum. Equimolar qu­anti­ties (1:1 stoichiometric ratio) of AKBA crystals and cinnamic acid were dissolved in hot ethanol to obtain a clear homogeneous solution. The resulting solution was then allowed to cool slowly and allowed slow evaporation to obtain crystals suitable for SXRD analysis.

## 6. Refinement

Crystal data, data collection and structure refinement details are summarized in Table 2 ▸. All hydrogen atoms were placed at idealized positions C—H = 0.96–0.98 Å) and refined using a riding model with *U*_iso_(H) = 1.2–1.5*U*_eq_(C). The assignment of the absolute configuration is based on IUPAC nomenclature.

## Supplementary Material

Crystal structure: contains datablock(s) I. DOI: 10.1107/S2056989026005360/ee2029sup1.cif

Structure factors: contains datablock(s) I. DOI: 10.1107/S2056989026005360/ee2029Isup3.hkl

CCDC reference: 2555762

Additional supporting information:  crystallographic information; 3D view; checkCIF report

## Figures and Tables

**Table 1:** Hydrogen-bond geometry (Å, °)

*D*—H⋯*A*	*D*—H	H⋯*A*	*D*⋯*A*	*D*—H⋯*A*
O4—H4⋯O1^i^	0.82	1.91	2.695 (4)	161
C1—H1*A*⋯O5	0.97	2.39	3.023 (5)	122
C25—H25*C*⋯O5	0.96	2.38	3.058 (5)	127
C30—H30*B*⋯O1^ii^	0.96	2.60	3.468 (6)	151
C32—H32*C*⋯O3^iii^	0.96	2.52	3.445 (6)	162

Symmetry codes: (i) 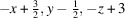; (ii) 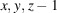; (iii) 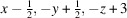.

**Table 2:** Experimental details

Crystal data	
Chemical formula	C_32_H_48_O_5_
*M*_r_	512.70
Crystal system, space group	Orthorhombic, *P*2_1_2_1_2
Temperature (K)	293
*a*, *b*, *c* (Å)	11.8995 (8), 16.3460 (9), 15.7065 (9)
*V* (Å^3^)	3055.0 (3)
*Z*	4
Radiation type	Mo *K*α
μ (mm^−1^)	0.07
Crystal size (mm)	0.26 × 0.24 × 0.22

Data collection	
Diffractometer	Rigaku **model?**
Absorption correction	Multi-scan (*CrysAlis PRO*; Rigaku OD, 2024 ▸)
*T*_min_, *T*_max_	0.981, 0.984
No. of measured, independent and observed [*I* > 2σ(*I*)] reflections	21419, 6775, 3256
*R*_int_	0.071
(sin θ/λ)_max_ (Å^−1^)	0.662

Refinement	
*R*[*F*^2^ > 2σ(*F*^2^)], *wR*(*F*^2^), *S*	0.058, 0.155, 0.95
No. of reflections	6775
No. of parameters	344
H-atom treatment	H-atom parameters constrained
Δρ_max_, Δρ_min_ (e Å^−3^)	0.17, −0.16
Absolute structure	Flack *x* determined using 990 quotients [(*I*^+^)−(*I*^−^)]/[(*I*^+^)+(*I*^−^)] (Parsons *et al.*, 2013 ▸)
Absolute structure parameter	0.6 (10)

Computer programs: *CrysAlis PRO* (Rigaku OD, 2024 ▸), *SHELXS* (Sheldrick, 2008 ▸), *SHELXL2018/3* (Sheldrick, 2015 ▸) and *OLEX2* (Dolomanov *et al.*, 2009 ▸).

## supplementary crystallographic information

### 3-Acetyl-11-keto-β-boswellic acid Crystal data

**Table:**

C_32_H_48_O_5_	*D*_x_ = 1.115 Mg m^−^^3^
*M**_r_* = 512.70	Mo *K*α radiation, λ = 0.71073 Å
Orthorhombic, *P*2_1_2_1_2	Cell parameters from 6775 reflections
*a* = 11.8995 (8) Å	θ = 1.8–28.1°
*b* = 16.3460 (9) Å	µ = 0.07 mm^−^^1^
*c* = 15.7065 (9) Å	*T* = 293 K
*V* = 3055.0 (3) Å^3^	Rock, white
*Z* = 4	0.26 × 0.24 × 0.22 mm
*F*(000) = 1120	

### 3-Acetyl-11-keto-β-boswellic acid Data collection

**Table:**

Rigaku model?diffractometer	3256 reflections with *I* > 2σ(*I*)
Multi–scan	*R*_int_ = 0.071
Absorption correction: multi-scan (CrysAlis PRO; Rigaku OD, 2024)	θ_max_ = 28.1°, θ_min_ = 1.8°
*T*_min_ = 0.981, *T*_max_ = 0.984	*h* = −15→13
21419 measured reflections	*k* = −21→20
6775 independent reflections	*l* = −16→20

### 3-Acetyl-11-keto-β-boswellic acid Refinement

**Table:**

Refinement on *F*^2^	H-atom parameters constrained
Least-squares matrix: full	*w* = 1/[σ^2^(*F*_o_^2^) + (0.0631*P*)^2^] where *P* = (*F*_o_^2^ + 2*F*_c_^2^)/3
*R*[*F*^2^ > 2σ(*F*^2^)] = 0.058	(Δ/σ)_max_ = 0.004
*wR*(*F*^2^) = 0.155	Δρ_max_ = 0.17 e Å^−^^3^
*S* = 0.95	Δρ_min_ = −0.16 e Å^−^^3^
6775 reflections	Extinction correction: SHELXL-2018/3 (Sheldrick 2015), Fc^*^=kFc[1+0.001xFc^2^λ^3^/sin(2θ)]^-1/4^
344 parameters	Extinction coefficient: 0.0054 (14)
0 restraints	Absolute structure: Flack *x* determined using 990 quotients [(*I*^+^)-(*I*^-^)]/[(*I*^+^)+(*I*^-^)] (Parsons *et al.*, 2013)
Hydrogen site location: inferred from neighbouring sites	Absolute structure parameter: 0.6 (10)

### 3-Acetyl-11-keto-β-boswellic acid Special details

**Table:**

Geometry. All esds (except the esd in the dihedral angle between two l.s. planes) are estimated using the full covariance matrix. The cell esds are taken into account individually in the estimation of esds in distances, angles and torsion angles; correlations between esds in cell parameters are only used when they are defined by crystal symmetry. An approximate (isotropic) treatment of cell esds is used for estimating esds involving l.s. planes.

### 3-Acetyl-11-keto-β-boswellic acid Fractional atomic coordinates and isotropic or equivalent isotropic displacement parameters (Å^2^)

**Table:**

	*x*	*y*	*z*	*U*_iso_*/*U*_eq_	
C18	0.8263 (3)	0.1344 (2)	0.8671 (2)	0.0647 (10)	
H18	0.904794	0.127092	0.849952	0.078*	
O2	0.6711 (2)	0.28353 (14)	1.38435 (16)	0.0754 (8)	
C9	0.8163 (3)	0.14879 (19)	1.1508 (2)	0.0547 (9)	
H9	0.774299	0.199366	1.140404	0.066*	
C5	0.7083 (3)	0.1303 (2)	1.2877 (2)	0.0584 (10)	
H5	0.663179	0.175629	1.265316	0.070*	
C13	0.8257 (3)	0.1324 (2)	0.9645 (2)	0.0581 (10)	
C10	0.8270 (3)	0.14465 (19)	1.2504 (2)	0.0565 (9)	
C11	0.9241 (3)	0.1600 (2)	1.1012 (3)	0.0635 (10)	
C7	0.6369 (3)	0.0635 (2)	1.1551 (2)	0.0654 (10)	
H7A	0.584322	0.107890	1.145585	0.079*	
H7B	0.603323	0.013902	1.132752	0.079*	
C3	0.7493 (4)	0.2204 (2)	1.4153 (3)	0.0691 (11)	
H3	0.753162	0.222089	1.477613	0.083*	
C6	0.6541 (3)	0.0534 (2)	1.2496 (2)	0.0678 (11)	
H6A	0.702046	0.006512	1.260158	0.081*	
H6B	0.582292	0.043524	1.276925	0.081*	
C12	0.9183 (3)	0.15086 (19)	1.0080 (3)	0.0621 (10)	
H12	0.984190	0.158588	0.977235	0.075*	
C4	0.6985 (3)	0.1387 (2)	1.3861 (2)	0.0656 (11)	
O4	0.7212 (3)	−0.00278 (16)	1.41652 (18)	0.1025 (11)	
H4	0.735364	−0.035366	1.454747	0.154*	
C1	0.8679 (3)	0.2302 (2)	1.2813 (3)	0.0677 (11)	
H1A	0.944279	0.238967	1.261724	0.081*	
H1B	0.820861	0.272144	1.256037	0.081*	
C16	0.6425 (3)	0.0608 (3)	0.8678 (3)	0.0859 (13)	
H16A	0.605178	0.111622	0.853010	0.103*	
H16B	0.599911	0.016317	0.842725	0.103*	
O5	1.0147 (2)	0.17837 (18)	1.13377 (17)	0.0879 (9)	
C8	0.7460 (3)	0.08150 (18)	1.1051 (2)	0.0555 (9)	
C2	0.8643 (4)	0.2384 (2)	1.3777 (3)	0.0761 (12)	
H2A	0.918751	0.201162	1.402420	0.091*	
H2B	0.886217	0.293623	1.393160	0.091*	
C15	0.6423 (4)	0.0511 (2)	0.9652 (3)	0.0806 (13)	
H15A	0.667898	−0.003545	0.979415	0.097*	
H15B	0.565750	0.056592	0.985766	0.097*	
C19	0.7893 (3)	0.2195 (2)	0.8309 (2)	0.0696 (11)	
H19	0.712948	0.230256	0.851441	0.083*	
C14	0.7174 (3)	0.1142 (2)	1.0119 (2)	0.0595 (9)	
C17	0.7617 (4)	0.0611 (2)	0.8298 (3)	0.0804 (12)	
O3	0.8236 (3)	0.08341 (17)	1.4943 (2)	0.0934 (10)	
C27	0.6491 (3)	0.1955 (2)	1.0140 (3)	0.0726 (11)	
H27A	0.699187	0.240449	1.024465	0.109*	
H27B	0.594032	0.192863	1.058563	0.109*	
H27C	0.612170	0.203254	0.960296	0.109*	
O1	0.7217 (3)	0.36657 (16)	1.4882 (2)	0.1078 (12)	
C23	0.7574 (4)	0.0711 (2)	1.4386 (3)	0.0768 (12)	
C26	0.8150 (3)	0.0004 (2)	1.1019 (3)	0.0733 (11)	
H26A	0.889530	0.011747	1.081631	0.110*	
H26B	0.778888	−0.037538	1.064171	0.110*	
H26C	0.819116	−0.022762	1.157962	0.110*	
C24	0.5746 (4)	0.1356 (2)	1.4149 (3)	0.0872 (13)	
H24A	0.531034	0.172939	1.381207	0.131*	
H24B	0.569626	0.150849	1.473828	0.131*	
H24C	0.546079	0.081116	1.407668	0.131*	
C25	0.9125 (3)	0.0795 (2)	1.2798 (3)	0.0734 (11)	
H25A	0.880435	0.025969	1.272972	0.110*	
H25B	0.930777	0.088241	1.338604	0.110*	
H25C	0.979502	0.083631	1.245995	0.110*	
C20	0.7854 (4)	0.2204 (3)	0.7323 (3)	0.0883 (13)	
H20	0.862199	0.211959	0.711537	0.106*	
C22	0.7544 (5)	0.0691 (3)	0.7322 (3)	0.1049 (16)	
H22A	0.828239	0.058615	0.708462	0.126*	
H22B	0.704266	0.026918	0.710992	0.126*	
C31	0.6617 (4)	0.3518 (2)	1.4287 (3)	0.0771 (12)	
C32	0.5735 (4)	0.4064 (2)	1.3955 (3)	0.0976 (14)	
H32A	0.563288	0.396468	1.335755	0.146*	
H32B	0.595476	0.462339	1.404077	0.146*	
H32C	0.504313	0.396088	1.425005	0.146*	
C29	0.8637 (4)	0.2875 (3)	0.8631 (3)	0.0955 (14)	
H29A	0.860074	0.289520	0.924169	0.143*	
H29B	0.838636	0.338766	0.840076	0.143*	
H29C	0.939856	0.277619	0.845714	0.143*	
C21	0.7141 (5)	0.1502 (4)	0.6997 (3)	0.1055 (16)	
H21A	0.636805	0.158426	0.717432	0.127*	
H21B	0.715825	0.149903	0.637999	0.127*	
C28	0.8231 (5)	−0.0187 (3)	0.8530 (3)	0.1089 (16)	
H28A	0.893397	−0.021101	0.823039	0.163*	
H28B	0.777492	−0.064642	0.837109	0.163*	
H28C	0.836816	−0.020016	0.913169	0.163*	
C30	0.7443 (5)	0.3010 (3)	0.6975 (3)	0.1197 (18)	
H30A	0.671966	0.313323	0.721263	0.179*	
H30B	0.738440	0.297557	0.636666	0.179*	
H30C	0.796512	0.343426	0.712480	0.179*	

### 3-Acetyl-11-keto-β-boswellic acid Atomic displacement parameters (Å^2^)

**Table:**

	*U*^11^	*U*^22^	*U*^33^	*U*^12^	*U*^13^	*U*^23^
C18	0.055 (2)	0.065 (2)	0.074 (3)	−0.0002 (19)	0.010 (2)	−0.0078 (19)
O2	0.095 (2)	0.0501 (14)	0.0807 (17)	0.0167 (14)	−0.0065 (15)	−0.0069 (13)
C9	0.047 (2)	0.0423 (18)	0.075 (3)	−0.0010 (16)	−0.0059 (19)	0.0048 (17)
C5	0.056 (2)	0.0433 (19)	0.076 (3)	−0.0002 (16)	−0.004 (2)	0.0051 (17)
C13	0.048 (2)	0.0461 (19)	0.081 (3)	0.0030 (17)	0.003 (2)	−0.0013 (18)
C10	0.050 (2)	0.0402 (18)	0.080 (3)	0.0024 (17)	−0.0068 (19)	0.0021 (17)
C11	0.044 (2)	0.057 (2)	0.090 (3)	−0.0059 (18)	−0.004 (2)	0.008 (2)
C7	0.055 (2)	0.060 (2)	0.081 (3)	−0.0107 (19)	0.000 (2)	0.0023 (19)
C3	0.088 (3)	0.050 (2)	0.069 (2)	0.010 (2)	−0.010 (2)	0.0003 (18)
C6	0.067 (3)	0.056 (2)	0.081 (3)	−0.009 (2)	0.003 (2)	0.0057 (19)
C12	0.044 (2)	0.062 (2)	0.080 (3)	−0.0035 (18)	0.005 (2)	0.004 (2)
C4	0.075 (3)	0.047 (2)	0.075 (3)	0.0000 (18)	−0.004 (2)	−0.0002 (19)
O4	0.160 (3)	0.0521 (16)	0.095 (2)	−0.0083 (18)	−0.029 (2)	0.0153 (15)
C1	0.062 (2)	0.055 (2)	0.087 (3)	−0.0079 (19)	−0.011 (2)	−0.002 (2)
C16	0.069 (3)	0.105 (3)	0.084 (3)	−0.026 (3)	−0.002 (2)	−0.018 (2)
O5	0.0552 (18)	0.111 (2)	0.097 (2)	−0.0199 (15)	−0.0107 (16)	0.0098 (17)
C8	0.043 (2)	0.0477 (18)	0.076 (2)	−0.0030 (16)	−0.0002 (19)	0.0029 (18)
C2	0.088 (3)	0.049 (2)	0.091 (3)	0.000 (2)	−0.027 (3)	−0.008 (2)
C15	0.061 (3)	0.097 (3)	0.084 (3)	−0.024 (2)	−0.001 (2)	−0.006 (2)
C19	0.061 (3)	0.077 (3)	0.071 (3)	0.004 (2)	0.005 (2)	0.003 (2)
C14	0.044 (2)	0.060 (2)	0.074 (2)	−0.0042 (17)	−0.0025 (19)	0.0024 (19)
C17	0.078 (3)	0.082 (3)	0.082 (3)	−0.013 (3)	0.011 (3)	−0.019 (2)
O3	0.120 (3)	0.0680 (18)	0.092 (2)	0.0050 (17)	−0.027 (2)	0.0057 (16)
C27	0.053 (2)	0.085 (3)	0.080 (3)	0.017 (2)	0.004 (2)	0.009 (2)
O1	0.176 (3)	0.0553 (16)	0.092 (2)	0.0202 (19)	−0.026 (2)	−0.0187 (16)
C23	0.103 (4)	0.046 (2)	0.081 (3)	0.005 (2)	0.001 (3)	0.005 (2)
C26	0.070 (3)	0.051 (2)	0.099 (3)	−0.0016 (18)	−0.003 (2)	−0.001 (2)
C24	0.089 (3)	0.076 (3)	0.097 (3)	0.001 (2)	0.022 (3)	−0.002 (2)
C25	0.067 (3)	0.063 (2)	0.090 (3)	0.012 (2)	−0.008 (2)	0.008 (2)
C20	0.087 (3)	0.101 (3)	0.076 (3)	−0.004 (3)	0.015 (3)	−0.005 (3)
C22	0.108 (4)	0.113 (4)	0.094 (4)	−0.029 (4)	0.014 (3)	−0.035 (3)
C31	0.107 (4)	0.049 (2)	0.076 (3)	0.001 (2)	0.006 (3)	−0.002 (2)
C32	0.109 (4)	0.062 (2)	0.122 (4)	0.024 (3)	0.010 (3)	0.006 (3)
C29	0.108 (4)	0.075 (3)	0.103 (3)	−0.009 (3)	−0.017 (3)	0.010 (2)
C21	0.114 (4)	0.129 (4)	0.074 (3)	−0.011 (4)	0.003 (3)	−0.006 (3)
C28	0.118 (4)	0.068 (3)	0.141 (4)	−0.005 (3)	0.005 (3)	−0.025 (3)
C30	0.146 (5)	0.122 (4)	0.091 (3)	0.018 (4)	0.002 (4)	0.027 (3)

### 3-Acetyl-11-keto-β-boswellic acid Geometric parameters (Å, º)

**Table:**

C18—H18	0.9800	C15—H15A	0.9700
C18—C13	1.530 (5)	C15—H15B	0.9700
C18—C19	1.566 (5)	C15—C14	1.549 (5)
C18—C17	1.539 (5)	C19—H19	0.9800
O2—C3	1.472 (4)	C19—C20	1.549 (6)
O2—C31	1.320 (5)	C19—C29	1.510 (5)
C9—H9	0.9800	C14—C27	1.558 (5)
C9—C10	1.571 (5)	C17—C22	1.541 (6)
C9—C11	1.511 (5)	C17—C28	1.539 (6)
C9—C8	1.558 (4)	O3—C23	1.195 (5)
C5—H5	0.9800	C27—H27A	0.9600
C5—C10	1.547 (5)	C27—H27B	0.9600
C5—C6	1.534 (5)	C27—H27C	0.9600
C5—C4	1.555 (5)	O1—C31	1.201 (5)
C13—C12	1.331 (5)	C26—H26A	0.9600
C13—C14	1.518 (5)	C26—H26B	0.9600
C10—C1	1.557 (4)	C26—H26C	0.9600
C10—C25	1.544 (5)	C24—H24A	0.9600
C11—C12	1.474 (5)	C24—H24B	0.9600
C11—O5	1.230 (4)	C24—H24C	0.9600
C7—H7A	0.9700	C25—H25A	0.9600
C7—H7B	0.9700	C25—H25B	0.9600
C7—C6	1.507 (5)	C25—H25C	0.9600
C7—C8	1.546 (5)	C20—H20	0.9800
C3—H3	0.9800	C20—C21	1.516 (7)
C3—C4	1.537 (5)	C20—C30	1.508 (6)
C3—C2	1.520 (6)	C22—H22A	0.9700
C6—H6A	0.9700	C22—H22B	0.9700
C6—H6B	0.9700	C22—C21	1.499 (7)
C12—H12	0.9300	C31—C32	1.473 (6)
C4—C23	1.546 (6)	C32—H32A	0.9600
C4—C24	1.543 (5)	C32—H32B	0.9600
O4—H4	0.8200	C32—H32C	0.9600
O4—C23	1.328 (5)	C29—H29A	0.9600
C1—H1A	0.9700	C29—H29B	0.9600
C1—H1B	0.9700	C29—H29C	0.9600
C1—C2	1.521 (5)	C21—H21A	0.9700
C16—H16A	0.9700	C21—H21B	0.9700
C16—H16B	0.9700	C28—H28A	0.9600
C16—C15	1.538 (5)	C28—H28B	0.9600
C16—C17	1.539 (6)	C28—H28C	0.9600
C8—C14	1.594 (5)	C30—H30A	0.9600
C8—C26	1.559 (5)	C30—H30B	0.9600
C2—H2A	0.9700	C30—H30C	0.9600
C2—H2B	0.9700		
			
C13—C18—H18	106.0	C20—C19—C18	112.3 (3)
C13—C18—C19	112.4 (3)	C20—C19—H19	107.5
C13—C18—C17	111.2 (3)	C29—C19—C18	111.6 (3)
C19—C18—H18	106.0	C29—C19—H19	107.5
C17—C18—H18	106.0	C29—C19—C20	110.2 (3)
C17—C18—C19	114.3 (3)	C13—C14—C8	109.5 (3)
C31—O2—C3	118.1 (3)	C13—C14—C15	112.8 (3)
C10—C9—H9	104.1	C13—C14—C27	106.6 (3)
C11—C9—H9	104.1	C15—C14—C8	109.6 (3)
C11—C9—C10	116.8 (3)	C15—C14—C27	106.1 (3)
C11—C9—C8	107.6 (3)	C27—C14—C8	112.2 (3)
C8—C9—H9	104.1	C18—C17—C22	110.0 (3)
C8—C9—C10	118.2 (3)	C16—C17—C18	108.3 (3)
C10—C5—H5	104.4	C16—C17—C22	109.5 (4)
C10—C5—C4	115.5 (3)	C28—C17—C18	109.4 (4)
C6—C5—H5	104.4	C28—C17—C16	110.1 (4)
C6—C5—C10	111.1 (3)	C28—C17—C22	109.5 (4)
C6—C5—C4	115.4 (3)	C14—C27—H27A	109.5
C4—C5—H5	104.4	C14—C27—H27B	109.5
C12—C13—C18	120.3 (3)	C14—C27—H27C	109.5
C12—C13—C14	119.7 (3)	H27A—C27—H27B	109.5
C14—C13—C18	119.9 (3)	H27A—C27—H27C	109.5
C5—C10—C9	108.0 (3)	H27B—C27—H27C	109.5
C5—C10—C1	107.7 (3)	O4—C23—C4	111.3 (4)
C1—C10—C9	107.2 (3)	O3—C23—C4	124.7 (4)
C25—C10—C9	112.3 (3)	O3—C23—O4	123.9 (4)
C25—C10—C5	112.6 (3)	C8—C26—H26A	109.5
C25—C10—C1	108.7 (3)	C8—C26—H26B	109.5
C12—C11—C9	117.4 (3)	C8—C26—H26C	109.5
O5—C11—C9	124.0 (4)	H26A—C26—H26B	109.5
O5—C11—C12	118.6 (4)	H26A—C26—H26C	109.5
H7A—C7—H7B	107.6	H26B—C26—H26C	109.5
C6—C7—H7A	108.7	C4—C24—H24A	109.5
C6—C7—H7B	108.7	C4—C24—H24B	109.5
C6—C7—C8	114.1 (3)	C4—C24—H24C	109.5
C8—C7—H7A	108.7	H24A—C24—H24B	109.5
C8—C7—H7B	108.7	H24A—C24—H24C	109.5
O2—C3—H3	109.9	H24B—C24—H24C	109.5
O2—C3—C4	105.2 (3)	C10—C25—H25A	109.5
O2—C3—C2	107.8 (3)	C10—C25—H25B	109.5
C4—C3—H3	109.9	C10—C25—H25C	109.5
C2—C3—H3	109.9	H25A—C25—H25B	109.5
C2—C3—C4	114.0 (3)	H25A—C25—H25C	109.5
C5—C6—H6A	109.5	H25B—C25—H25C	109.5
C5—C6—H6B	109.5	C19—C20—H20	107.7
C7—C6—C5	110.6 (3)	C21—C20—C19	110.3 (4)
C7—C6—H6A	109.5	C21—C20—H20	107.7
C7—C6—H6B	109.5	C30—C20—C19	112.3 (4)
H6A—C6—H6B	108.1	C30—C20—H20	107.7
C13—C12—C11	124.9 (3)	C30—C20—C21	111.0 (4)
C13—C12—H12	117.6	C17—C22—H22A	108.4
C11—C12—H12	117.6	C17—C22—H22B	108.4
C3—C4—C5	110.1 (3)	H22A—C22—H22B	107.5
C3—C4—C23	106.5 (3)	C21—C22—C17	115.5 (4)
C3—C4—C24	108.5 (3)	C21—C22—H22A	108.4
C23—C4—C5	115.6 (3)	C21—C22—H22B	108.4
C24—C4—C5	111.1 (3)	O2—C31—C32	112.7 (4)
C24—C4—C23	104.7 (3)	O1—C31—O2	122.0 (4)
C23—O4—H4	109.5	O1—C31—C32	125.3 (4)
C10—C1—H1A	109.1	C31—C32—H32A	109.5
C10—C1—H1B	109.1	C31—C32—H32B	109.5
H1A—C1—H1B	107.9	C31—C32—H32C	109.5
C2—C1—C10	112.4 (3)	H32A—C32—H32B	109.5
C2—C1—H1A	109.1	H32A—C32—H32C	109.5
C2—C1—H1B	109.1	H32B—C32—H32C	109.5
H16A—C16—H16B	107.8	C19—C29—H29A	109.5
C15—C16—H16A	109.0	C19—C29—H29B	109.5
C15—C16—H16B	109.0	C19—C29—H29C	109.5
C15—C16—C17	112.8 (4)	H29A—C29—H29B	109.5
C17—C16—H16A	109.0	H29A—C29—H29C	109.5
C17—C16—H16B	109.0	H29B—C29—H29C	109.5
C9—C8—C14	107.6 (2)	C20—C21—H21A	109.2
C9—C8—C26	109.4 (3)	C20—C21—H21B	109.2
C7—C8—C9	110.5 (3)	C22—C21—C20	112.1 (4)
C7—C8—C14	110.6 (3)	C22—C21—H21A	109.2
C7—C8—C26	107.3 (3)	C22—C21—H21B	109.2
C26—C8—C14	111.6 (3)	H21A—C21—H21B	107.9
C3—C2—C1	113.3 (3)	C17—C28—H28A	109.5
C3—C2—H2A	108.9	C17—C28—H28B	109.5
C3—C2—H2B	108.9	C17—C28—H28C	109.5
C1—C2—H2A	108.9	H28A—C28—H28B	109.5
C1—C2—H2B	108.9	H28A—C28—H28C	109.5
H2A—C2—H2B	107.7	H28B—C28—H28C	109.5
C16—C15—H15A	108.8	C20—C30—H30A	109.5
C16—C15—H15B	108.8	C20—C30—H30B	109.5
C16—C15—C14	113.7 (3)	C20—C30—H30C	109.5
H15A—C15—H15B	107.7	H30A—C30—H30B	109.5
C14—C15—H15A	108.8	H30A—C30—H30C	109.5
C14—C15—H15B	108.8	H30B—C30—H30C	109.5
C18—C19—H19	107.5		
			
C18—C13—C12—C11	172.7 (3)	C6—C7—C8—C9	−46.1 (4)
C18—C13—C14—C8	157.9 (3)	C6—C7—C8—C14	−165.0 (3)
C18—C13—C14—C15	35.6 (4)	C6—C7—C8—C26	73.1 (4)
C18—C13—C14—C27	−80.4 (4)	C12—C13—C14—C8	−25.8 (4)
C18—C19—C20—C21	52.9 (5)	C12—C13—C14—C15	−148.1 (3)
C18—C19—C20—C30	177.2 (4)	C12—C13—C14—C27	95.9 (4)
C18—C17—C22—C21	−49.9 (6)	C4—C5—C10—C9	−170.3 (3)
O2—C3—C4—C5	70.1 (4)	C4—C5—C10—C1	−54.8 (4)
O2—C3—C4—C23	−163.9 (3)	C4—C5—C10—C25	65.0 (4)
O2—C3—C4—C24	−51.7 (4)	C4—C5—C6—C7	162.7 (3)
O2—C3—C2—C1	−65.0 (4)	C4—C3—C2—C1	51.4 (4)
C9—C10—C1—C2	171.1 (3)	C16—C15—C14—C13	−38.0 (5)
C9—C11—C12—C13	−1.0 (5)	C16—C15—C14—C8	−160.3 (3)
C9—C8—C14—C13	58.6 (3)	C16—C15—C14—C27	78.4 (4)
C9—C8—C14—C15	−177.2 (3)	C16—C17—C22—C21	69.0 (5)
C9—C8—C14—C27	−59.6 (3)	O5—C11—C12—C13	−179.0 (3)
C5—C10—C1—C2	55.1 (4)	C8—C9—C10—C5	−47.2 (4)
C5—C4—C23—O4	−55.8 (5)	C8—C9—C10—C1	−163.0 (3)
C5—C4—C23—O3	127.1 (5)	C8—C9—C10—C25	77.6 (4)
C13—C18—C19—C20	−177.8 (3)	C8—C9—C11—C12	34.8 (4)
C13—C18—C19—C29	57.9 (4)	C8—C9—C11—O5	−147.3 (3)
C13—C18—C17—C16	55.1 (4)	C8—C7—C6—C5	58.0 (4)
C13—C18—C17—C22	174.8 (4)	C2—C3—C4—C5	−47.8 (4)
C13—C18—C17—C28	−64.9 (4)	C2—C3—C4—C23	78.3 (4)
C10—C9—C11—C12	170.5 (3)	C2—C3—C4—C24	−169.6 (3)
C10—C9—C11—O5	−11.6 (5)	C15—C16—C17—C18	−61.3 (5)
C10—C9—C8—C7	42.1 (4)	C15—C16—C17—C22	178.7 (3)
C10—C9—C8—C14	162.9 (3)	C15—C16—C17—C28	58.3 (5)
C10—C9—C8—C26	−75.8 (4)	C19—C18—C13—C12	−91.8 (4)
C10—C5—C6—C7	−63.3 (4)	C19—C18—C13—C14	84.5 (4)
C10—C5—C4—C3	51.6 (4)	C19—C18—C17—C16	−73.6 (4)
C10—C5—C4—C23	−69.1 (4)	C19—C18—C17—C22	46.1 (5)
C10—C5—C4—C24	171.8 (3)	C19—C18—C17—C28	166.4 (4)
C10—C1—C2—C3	−55.2 (4)	C19—C20—C21—C22	−55.9 (5)
C11—C9—C10—C5	−178.2 (3)	C14—C13—C12—C11	−3.6 (5)
C11—C9—C10—C1	66.0 (4)	C17—C18—C13—C12	138.5 (4)
C11—C9—C10—C25	−53.4 (4)	C17—C18—C13—C14	−45.2 (4)
C11—C9—C8—C7	177.0 (3)	C17—C18—C19—C20	−49.7 (5)
C11—C9—C8—C14	−62.2 (3)	C17—C18—C19—C29	−174.1 (4)
C11—C9—C8—C26	59.2 (4)	C17—C16—C15—C14	53.2 (5)
C7—C8—C14—C13	179.3 (3)	C17—C22—C21—C20	56.2 (6)
C7—C8—C14—C15	−56.4 (4)	C26—C8—C14—C13	−61.4 (3)
C7—C8—C14—C27	61.1 (3)	C26—C8—C14—C15	62.8 (4)
C3—O2—C31—O1	6.8 (6)	C26—C8—C14—C27	−179.6 (3)
C3—O2—C31—C32	−174.7 (3)	C24—C4—C23—O4	66.8 (4)
C3—C4—C23—O4	−178.4 (4)	C24—C4—C23—O3	−110.4 (5)
C3—C4—C23—O3	4.4 (6)	C25—C10—C1—C2	−67.2 (4)
C6—C5—C10—C9	55.8 (3)	C31—O2—C3—C4	149.8 (3)
C6—C5—C10—C1	171.3 (3)	C31—O2—C3—C2	−88.2 (4)
C6—C5—C10—C25	−68.9 (4)	C29—C19—C20—C21	178.0 (4)
C6—C5—C4—C3	−176.5 (3)	C29—C19—C20—C30	−57.7 (5)
C6—C5—C4—C23	62.8 (4)	C28—C17—C22—C21	−170.1 (5)
C6—C5—C4—C24	−56.3 (4)	C30—C20—C21—C22	179.0 (4)

### 3-Acetyl-11-keto-β-boswellic acid Hydrogen-bond geometry (Å, º)

**Table:**

*D*—H···*A*	*D*—H	H···*A*	*D*···*A*	*D*—H···*A*
O4—H4···O1^i^	0.82	1.91	2.695 (4)	161
C1—H1*A*···O5	0.97	2.39	3.023 (5)	122
C25—H25*C*···O5	0.96	2.38	3.058 (5)	127
C30—H30*B*···O1^ii^	0.96	2.60	3.468 (6)	151
C32—H32*C*···O3^iii^	0.96	2.52	3.445 (6)	162

Symmetry codes: (i) −*x*+3/2, *y*−1/2, −*z*+3; (ii) *x*, *y*, *z*−1; (iii) *x*−1/2, −*y*+1/2, −*z*+3.
